# Reality in the 1990s and beyond—more with less

**DOI:** 10.1155/S1463924695000150

**Published:** 1995

**Authors:** Philip A. Lane

**Affiliations:** Director Analytical Research and Development R.W. Johnson Pharmaceutical Research Institute Raritan New Jersey USA

## Abstract

It is no secret that the pharmaceutical industry is undergoing rapid and revolutionary change. The impact of managed care and threatened pricing caps by the US Congress on new products has caused many companies to re-evaluate their short and long term business and research strategies. In too many cases, this has resulted in down-sizing by both lay-offs and attrition. For most analytical laboratories, this has meant doing more with less resources. There are several major ‘realities’ that are having a significant influence on the amount and type of analytical support required to bring a new product to the market place in today's regulatory climate. They are pre-approval inspections, the Barr decision and the proposed ICH guidelines. Other realities of the 1990s are also influencing the operation of the analytical laboratory. To cope with these realities, wise use of resources is mandatory. The strategies employed by each company differ, but laboratory automation is usually one of the important elements of the equation. Other elements include contract laboratories, consultants and temporaries. Each of these elements provides part of the solution to doing more with less, but each has its own positives and negatives which must be considered. This paper looks at the relationships between these factors and their impact on the analytical laboratory.


Journal of Automatic Chemistry, Vol. 17, No. 3 (May-June 1995), pp. 89-93

### Reality in the 1990s and beyond—more with less

**Philip A. Lane**

*Director, Analytical Research and Development, R.W. Johnson Pharmaceutical Research Institute, Raritan, New Jersey, USA*

*It is no secret that the pharmaceutical industry is undergoing rapid and revolutionary change. The impact of managed care and threatened pricing caps by the US Congress on new products has caused many companies to re-evaluate their short and long term business and research strategies. In too many cases, this has resulted in down-sizing by both lay-offs and attrition. For most analytical laboratories, this has meant doing more with less resources. There are several major 'realities' that are having a significant influence on the amount and type of analytical support required to bring a new product to the market place in today's regulatory climate. They are pre-approval inspections, the Barr decision and the proposed ICH guidelines. Other realities of the 1990s are also influencing the operation of the analytical laboratory. To cope with these realities, wise use of resources is mandatory. The strategies employed by each company differ, but laboratory automation is usually one of the important elements of the equation. Other elements include contract laboratories, consultants and temporaries. Each of these elements provides part of the solution to doing more with less, but each has its own positives and negatives which must be considered. This paper looks at the relationships between these factors and their impact on the analytical laboratory.*

### Introduction

Forty per cent of this decade is already in the history books. For those of us in the health care business and other related industries the 'realities' of the 1990s and beyond are coming into focus. Unfortunately, the picture is not always encouraging or reassuring. Too many times we hear or read about different companies reducing their payroll, or divesting themselves of unprofitable businesses, or merging, or restructuring. Regardless of the words used, the work force is usually being decreased. We also hear and read about new regulations being imposed on business by the local, state and federal governments. Many of these new regulations increase the cost of goods. The cost increase is always passed on to us, the consumer, pushing up the cost of living. That is reality!

I have been in the pharmaceutical industry for over 30 years and never has the attack on the pharmaceutical industry been as intense as it is now. To be opposed to the pharmaceutical industry is politically correct. In autumn 1994, the US Congress shelved voting on a health care bill until at least 1995. What impact a health care bill will have on drug prices and health care costs is uncertain, but, if history has taught us anything, once Washington gets involved things get worse and the costs go up.

Three of the biggest realities (figure 1) currently having an impact on the pharmaceutical industry are pre-approval inspections, the Barr decision and the ICH guidelines.

Pre-approval inspections
Barr decision
ICH guidelines

*Figure 1. Realities for the 1990s and beyond.*

### Realities of the 1990s

*Pre-approval inspections*

The generic drug scandal greatly increased the number of pre-approval inspections by the FDA in the USA. The legal authority to conduct these inspections is well-established by the FD&C Act and by the Agency's policies (figure 2). The objectives of Policy 7346.832 [1] are shown in figure 3. Essentially, the FDA wants to be sure that the firm adheres to cGMPs, can manufacture the product as described in the NDA, has accurate data on the bio-batches and can verify the firm's results themselves. FDA wants to do this within 45 days of accepting the NDA for filing (figure 4). I do not know if FDA has actually inspected anyone within 45 days, but they have within 90 days. In my company, this has had a great impact. It is our policy to have completed process validation reports within the 45-day window on three full-scale batches for all manufacturing sites that are

Section 704(a)(1) of FD&C Act
Compliance policy guide No. 7346.832—
issued October 1990
Generic drug scandal increased the number of
pre-approval inspections

*Figure 2. FDA legal authority for pre-approval inspection.*

Evaluation of firm's compliance to
cGMP's, including specific batches
used to demonstrate bioequivalence
(biobatches)
Evaluation if firm has adequate
facilities, equipment, procedures
and controls to manufacture product
as described in the application
Audit the accuracy of the biobatch
manufacturing and testing information
submitted in the application
Collection of a sample of the biobatch
from the bioequivalence test laboratory

*Figure 3. Objectives of CP 7346.832.*

© Zymark Corporation 1995

Have a field pre-approval inspection
within 45 days of acceptance of the
NDA filing by the FDA

*Figure 4. FDA goal.*

included in the NDA. Five years ago we used to do this about six months before we expected FDA approval of the NDA. This creates a lot of routine assay work and requires a significant commitment of resources much earlier than in the past.

The FDA's expectations during the inspection are shown in figure 5. These expectations are not unreasonable, yet we know that a significant number of firm's applications have not been approved as a result of the pre-approval inspection. I can't emphasize enough the need for good documentation. The FDA wants to see documentation. Can you prove you did it in accordance with your SOPs?

The FDA's approach to the inspection is spelled out in the FDA's Laboratory Inspection Guide [2], which was published in June 1992. Some of the areas that will be inspected are shown in figure 6.

One important area is the increased scrutiny that the FDA is giving to methods validation. According to the June 1994 issue of *The Gold Sheet* [3] about one-third of recent drug GMP warning letters have cited concerns involving the use of analytical methods. Besides inadequate methods validation, the FDA is also citing companies on how they handle records, procedures, equipment, reference standards and samples.

Firms provide adequate assurances that
drugs have the following features that they
purport to have
Identity
Quality
Purity
Strength
Good science used
Complete documentation available

*Figure 5. FDA expectations.*

Bulk drug substances
Microbiological data
Sampling
Laboratory records and documentation
Laboratory standard solutions
Methods validation
Equipment
Raw material testing
In-process control and specifications
Quality of data
Stability
Sampling protocols
Computerized laboratory data acquisition systems
Laboratory management
*Laboratory Inspection Guide* (22 June, 1992)

*Figure 6. Inspection approach.*

*The Barr decision*

On February 4th, 1993 Judge Wolin ruled on the case of the USA versus Barr Laboratories. Essentially Judge Wolin interpreted the GMPs. Some of the key areas he interpreted are shown on figure 7. The most controversial areas are probably retesting, resampling, averaging and using outlier tests to reject data. Judge Wolin essentially issued a set of rules about how out-of-specification laboratory results are to be handled. Out-of-specification results were divided into three classes (figure 8) documentable laboratory error, non-process related or operator error and process related or manufacturing process error. Laboratory error is the easiest to deal with and requires only an informal investigation. The other two, however, require a formal investigation and involve other areas, since the cause may be due to process issues. As a result, retesting or resampling cannot be automatically performed. In fact, he stated that resampling is always suspect. Defendable, documented reasons and decisions are required to retest and resample. The use of scientific judgement in the decision-making process is discussed by Judge Wolin. The decisions resulting from retesting and resampling must be documented and approved by the 'appropriate' level of management. Some FDA personnel mean your CEO, because they will hold him/her accountable.

This decision has a big impact on the operation of the analytical laboratory. Any GMP/GLP activity is covered by the Barr decision. Other activities are not. The FDA will tell you that work on developmental compounds are excluded, but, drug safety supplies, clinical supplies and process validation samples are. The result is that everyone in the laboratory has to be aware of the type of sample they are assaying and, if an out-of-specification result is

Out-of-specification (OOS) laboratory results
USP standards
Retesting
Resampling
Averaging assay results
Methods validation
Outlier statistical tests
Blend testing
Product release
Remixing
Validation criteria
Cleaning validation

*Figure 7. Barr decision.*

Three possible categories:
(1) Laboratory error
Analyst error with assignable cause
Informal investigation and documentation
(2) Non-process related or operator error
(3) Process related or manufacturing process error
(2) and (3) require formal investigation outside of the laboratory and formal documentation of findings and decisions

*Figure 8. Out-of-specification laboratory results.*

Harmonization of stability requirements
Detection and control of lower levels of
impurities and degradation products
0·1% or 1 mg/day intake (whichever is less)
if less than 2 g/day
0·05% if more than 2 g/day
Faster submissions
File with one year stability data
at 30°C/60% RH

*Figure 9. ICH guidelines.*

obtained, the correct response occurs. My advice is to have an SOP, because the FDA is looking to see how you handle this. The FDA has taken the Judge's ruling and is using it when they inspect.

If you have not read the entire 88 page decision, you should. It is very interesting and easy to read. You might read about something you are doing which the Judge has interpreted as being unacceptable.

*ICH guidelines*

Another reality facing the pharmaceutical analyst is the proposed ICH guidelines (see figure 9). The goal of the ICH guidelines is to harmonize submission requirements between the USA, Europe and Japan.

The stability guidelines [4] have essentially been agreed to. You will be able to file with 12 months at 30°C/60% RH data. This will put the analytical laboratory on the critical path to complete these assays as quickly as possible. The guidelines [5] on impurities and degradation products will also require that more sensitive and rugged methods be used. The proposed guideline states that, if 2 g or less of a drug is taken daily, then the impurities and degradation products must be controlled at the 0·1% level. If the daily dosage exceeds 2 g, then the impurities and degradation products must be controlled at the 0·05% level. In most cases this will mean that more sophisticated methods will be employed, requiring a highly trained staff, more time and more money.

### Additional realities of the 1990s

These three realities by themselves will require more resources to get the job done with the quality and speed that top management wants. Besides the three realities already discussed, there are several more realities that face us through the rest of the decade and into the 21st century. These are shown in figure 10.

Down-sizing and mergers
Diverse work force
Work and family programmes
IQ/OQ initiatives

*Figure 10. More realities for the 1990s.*

Do more with less—Faster
Age of unreason:
Core workers
Consultants
Temporaries
Contract services

*Figure 11. Down-sizing and mergers.*

*Down-sizing and mergers*

All too frequently the media tell us about companies down-sizing, about mergers, about re-structuring and about early voluntary retirement programmes (figure 11). There is a very high probability that we all will experience this during our careers. The annual chemist's salary survey published in the 11 July issue of *Chemical And Engineering News* [6] says that the job market for chemists in the USA is the weakest in 20 years. Salary gains also dipped.

The big challenge is how to manage an increasing workload with decreasing work staff. Some companies have increased their use of contract laboratories. Some have increased their use of temporaries and consultants. Many companies are doing both. In 1990, Professor Charles Handy of the Harvard Business School published a book, entitled *The Age Of Unreason* [7]. In this book Professor Handy describes the company of the future. It will consist of a small group of core workers who conduct the company business using contract firms, temporaries and consultants. The good news for people like us is that the technical and scientific staff are considered core workers by Professor Handy. The core workers would actually be employees of the company, but the temporaries and consultants would be self-employed and would work for the company only when the company needed them. An article in the 6 March *New York Times* [8] agreed with this. Professor Handy says this is the future, but, in my company and in many others, this is happening right now. The future is now.

Professor Handy also said that most people's careers at a company would end at about age 50. This would allow for young people with new ideas to join the company as core workers. Maybe the voluntary early retirement programmes are the first sign of this prediction. Nonetheless, the challenge is to do more with less—faster.

*Diverse work force*

The demographics say that the composition of the work force is changing (figure 12). The latest analysis of the composition of the United States work force shows that the number of women and minorities in science is slowly increasing. According to an article in *Today's Chemist At Work* [10] by the year 2000 white males will only be 49% of the work force. The way we work and how we think about work is changing. The work ethic is different in different cultures and the successful manager has to learn how work in the new environment. The manager will have to promote team work among diverse workers. How the manager does this will be scrutinized to be sure that the manager is not biased. So, the manager will have to

Work force is changing:
- By 2000 white males less than 50% of work force
- 20% increase in Hispanics in science since 1991
- Women represent 10% of science work force since 1991

Work ethic is changing
Teamwork

*Figure 12. Diverse work force.*

be able to effectively use the diverse work force to meet the company's goals within the established time line.

*Work and family programmes*

Work and family programmes (figure 13) are excellent programmes that help everyone cope with family needs. They do present challenges, however. Programmes, such as flex time and flex place, as helpful as they are, can be abused. With flex time, it is difficult sometimes to know that the employee worked a full day. With flex place the manager has to trust the employee is working his/her full day at home. When headcount is being reduced, or is remaining constant, and the workload is increasing, the potential loss in productivity created by flex time and place can be problematic if deadlines are crucial. Programmes for child care and care for aged parents also present problems for the manager. A sick child or parent can legitimately require an employee to be absent, but can cause loss of productivity.

The New Jersey Clean Air Act is an example of a state-mandated programme to reduce the number of single passenger automobiles on the road during rush hour, by forcing car pooling. This means the employee will leave when their car pool leaves, even if the employee has not completed their day's assignments. I wonder how productivity will suffer when this becomes law in 1996?

*IQ/OQ initiatives*

All of the various qualification initiatives (figure 14) are becoming reality. The FDA wants documentation to prove that the instruments are functioning as expected. My regulatory group wants me to write an installation qualification protocol to document that the new instrument was installed correctly and meets the vendor's specifications. If I had enough staff, I might entertain this request. I am not convinced that this adds value to the data. I think that there are better ways to ensure the quality and integrity of the data.

Flex time
Flex place
Child care
Care for aged parents
Federal Clean Air Act

*Figure 13. Work and family programmes.*

Installation qualification (IQ)
Operations qualification (OQ)
IQ—process of demonstrating instrument performs its intended functions within its specified tolerances
OQ—process of demonstrating a sequence of analytical operations behaves as expected

*Figure 14. IQ/OQ initiatives.*

Where are we going with all of these initiatives? Are we going to have an instrument check list that the chemist will initial for every step he performs? One of my regulatory colleagues keeps telling me that there will be analytical 'batch' sheets, similar to a manufacturing batch record, where every step the chemist performs will be verified by a second individual.

### Survival in the 1990s and beyond

Each of these realities presents formidable challenges to being efficient and highly productive for the remainder of the decade and into the next century. In fact your very survival as an organization may depend on how you respond to these realities. So, how are we going to survive? How are we going to do more with less resources? How are we going to do it faster? If Professor Handy is correct, we certainly will be using consultants, temporaries and contract service organizations to help us accomplish our objectives.

Decision-making will be crucial to survival. Management will have to make hard decisions about projects and stick to them. No organization will be able to afford spending resources on projects that do not have significant return on the investment. For example, probably no pharmaceutical company will be able to successfully market a number four 'me-too' product in the managed care environment we are entering. In the past, companies could do this and make big profits. Companies will have to carefully monitor their development portfolios and terminate projects if market forces require it.

Data flow will be crucial to survival. The most important product that the analytical chemist produces is accurate, reproducible data. If the data do not get to the right person at the right time no decision or, even worse, a bad decision could be made. LIMS will become very important to the organization. The company that can gather, process and evaluate data quickly will have a market advantage over the company that cannot, especially if they compete in the same markets. In my company we are implementing a new LIMS that will connect all of our worldwide sites so that we can instantly share data. This will allow us to make better decisions faster and will shorten cycle time for worldwide regulatory submissions and inquiries.

Finally, automation will be crucial to survival. I do not need to preach about the value of robotics in this journal. I will not cite productivity data. However, I will call your attention to an editorial in the June 1994 issue of *Pharmaceutical Technology* [11] by Francis Zenie, President of Zymark. In this excellent editorial Mr Zenie discusses

1. Increases capacity for significantly higher sample loads and more complex testing on each sample
2. Provides just-in-time analysis for faster new product introduction, timely correction of quality control problems and enhanced customer service.
3. Enhances precision, documentation and defensible audit trails.
4. Reduces analysis cost while routinely gathering all the data necessary to solve problems quickly—rather than wait for more data.
5. Transfers valid analytical methods to multiple sites worldwide.
6. Improves motivation, reduces turnover and enhances the effectiveness of a down-sized work force.
7. Utilizes valuable laboratory space more effectively.

*Figure 15. Francis Zenie's seven benefits of highly effective laboratory automation.*

'The seven benefits of highly effective laboratory automation'. The seven benefits are shown in figure 15. The seven benefits provide some of the answers to how to do more with less—faster.

### Summary

We can look at the realities we face and be pessimistic about the future or we can look at them as an opportunity. I look at them and see opportunities and challenges. Opportunities and challenges that will make us extend ourselves, make us think outside of the box, make us challenge the conventional thinking, make us go where no one has gone before. Things will change, so let us shape the realities of the future and not let someone else shape it for us.

### References

1. FDA Policy 7346.832 (October 1990).
2. FDA *Laboratory Inspection Guide* (22 June 1992).
3. *The Gold Sheet*, **28** (June 1994).
4. ICH Guidelines, *Stability Testing Of New Drug Substances And Products, Step 4* (27 October 1993).
5. ICH Guidelines, *Impurities in New Drug Substances, Draft #6* (15 March 1994).
6. *Chemical & Engineering News* (11 July 1994), p. 8.
7. Handy, C., *The Age Of Unreason* Harvard Business School Press (1990).
8. *New York Times*, Financial Section (Sunday, 6, March 1994).
9. Sankaran, N., *The Scientist* (7 March 1994), p. 3.
10. Borchardt, J. K., *Today's Chemist At Work*, **3**, 14 (1994).
11. Zenie, F., *Pharmaceutical Technology* (June 1994), p. 8.

